# An 18-Year-Old Female Experiences Unilateral Vocal Cord Paralysis during Mild COVID-19 Infection

**DOI:** 10.1155/2022/6059487

**Published:** 2022-07-08

**Authors:** John M. Coggins, Charisse Wright, Michael P. Underbrink

**Affiliations:** ^1^School of Medicine, University of Texas Medical Branch at Galveston, Galveston, Texas, USA; ^2^Houston ENT & Allergy, 915 Gessner, Suite 280, Houston, Texas 77024, USA

## Abstract

The COVID-19 pandemic has shown with certainty that SARS-CoV-2 can cause a variety of clinical findings, with some of the most notable being lasting chemosensory changes. Severe infections with SARS-CoV-2 can also lead to a variety of complications. For example, vocal cord paralysis can be caused by trauma sustained during intubation, which is a necessary procedure for many severe cases. Rarely, SARS-CoV-2 related vocal cord paralysis has occurred outside the context of intubation. These cases contribute to an emerging assortment of evidence supporting the neuropathic capacity of SARS-CoV-2. This report documents a case of COVID-19 related vocal cord paralysis in an 18-year-old female. The patient had a significant history of muscle tension dysphonia, chronic laryngitis, and vocal cord nodules. The patient developed vocal cord paralysis concurrently with the onset of mild viral symptoms and was never intubated or hospitalized. Based on the onset of symptoms and other causes being excluded with CT, a diagnosis of COVID-19-related vocal cord paralysis was performed.

## 1. Introduction

Vocal cord paralysis (VCP) presents with dysphonia, swallowing difficulties, and shortness of breath [1]. The severity can vary and is dangerously increased if paralysis is bilateral. Unilateral paralysis is more commonly left-sided, given the extended journey of the left recurrent laryngeal nerve and a resulting increase in opportunities for injuries to occur [2]. VCP is traditionally considered to have three primary etiologies: trauma, malignancy, and idiopathic causes [3]. The relative percentages of these etiologies have changed over time. Data from 1985–1995 in a large case series purported that malignancy was the most common cause of unilateral VCP [4]. The following decade, data shifted to support iatrogenic injury (37%) as the most common cause [4]. Currently, idiopathic VCP is the most common and many suspect that viral infections may be the primary culprit behind the unexplained loss of cord mobility. In these cases, the VCP is usually transient, difficult to distinguish from other inflammatory conditions, and may not be accompanied by traditional viral symptoms. For these reasons, the incidence and prevalence of viral VCP are difficult to determine. The varicella-zoster virus is the most commonly reported viral cause of VCP; however, many viruses have been implicated [5]. Patients with VCP are watched for 6–9 months for spontaneous recovery before surgical treatment is considered. Surgical approaches vary, but the common aim is to medialize the vocal fold and improve voice quality.

During the COVID-19 pandemic, intubation of patients with severe SARS-CoV-2 infections has been frequently necessary. Intubation is capable of causing trauma-related VCP due to the inflation of the cuff within the larynx [6]. Patients with prolonged intubation or difficult airways are particularly susceptible to the development of VCP [6]. On rare occasions, SARS-CoV-2-related VCP has been reported in the absence of intubation with only 6 cases reported in the literature as of September 2021 [7]. In this report, we will document the appearance of VCP in a relatively mild SARS-CoV-2 infection. The patient was not hospitalized or intubated, eliminating the possibility for a trauma-related etiology, and no evidence of malignancy was found. A few cases of VCP related to SARS-CoV-2 infections have been found in patients not intubated, making this case quite unusual. Moreover, this case documents the youngest patient to develop COVID-19-related VCP currently in the literature.

## 2. Case Presentation

**Figure 1:**
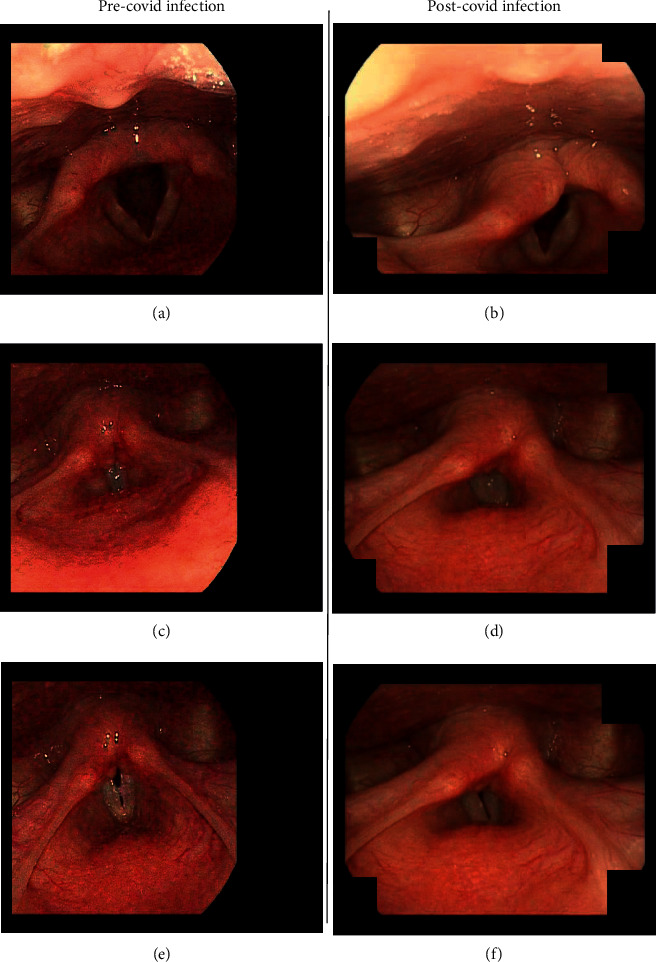
Videostroboscopy. A comparison of images taken 7 months before the SARS-CoV-2 infection (a, c, e) with images taken 2 months after the SARS-CoV-2 infection (b, d, f). Images (a, b) show abduction, images (c, d) show adduction, and images (e, f ) show relaxed phonation. Previous videostroboscopy was available for comparison due to prior evaluation of the patient's muscle tension dysphonia and vocal cord nodules.

An 18-year-old female with a history of vocal cord nodules, chronic laryngitis, gastroesophageal reflux disease (GERD), and muscle tension dysphonia presented with a two-month history of acute dysphonia. Two months prior to arrival, the patient became infected with the SARS-CoV-2 virus verified by a positive rapid antigen test. The patient experienced symptoms of headache, fatigue, muscle aches, cough, and acute dysphonia during her infection. All symptoms resolved after 10 days except for the acute dysphonia. The infection was treated at home with over-the-counter acetaminophen and ibuprofen. On presentation, her voice was slowly returning; however, she was having trouble projecting and continued to have dysphonia. The patient was experiencing daily aspiration episodes if she was not cautious while drinking liquids. Videostroboscopy revealed findings consistent with right true vocal fold paralysis (Figure 1), and a CT scan of the neck found no evidence of occult malignancies or the mass effect that could have caused the paralysis.

The patient's voice history is significant for prior vocal cord dysfunction. At 13 years of age, the patient was seen for persistent hoarseness accompanied by nasal congestion and GERD. Laryngoscopy revealed bilateral erythema of the vocal cords and a small nodule on the right vocal cord. The patient was diagnosed with chronic laryngitis caused by overuse and reflux disease and was prescribed reflux medications, speech therapy, and steroids. At 16 years of age, the patient returned to be seen for hoarseness. The patient described her voice as low-pitched, scratchy, strained, and effortful. Nasal congestion and heartburn were also noted in the patient's review of systems. The patient was prescribed reflux medications and vocal rest and was referred to a laryngologist for speech evaluation. Upon referral to the voice clinic, the laryngologist noted that the patient complained of a raspy voice since childhood due to her being a “loud talker” and frequently overusing her voice. The laryngologist described her voice as rough, breathy, and strained. The patient's voice received a 20/40 on the Voice Handicap Index (VHI). Laryngeal function studies were performed, and the patient was noted to have decreased breath support and a significantly strained voice consistent with muscle tension dysphonia. Videostroboscopy revealed nodules on the left and right vocal cords, an hourglass glottic closure, and anteroposterior compression. The patient was diagnosed with normal vocal mobility, muscle tension dysphonia, and vocal cord nodules, and the patient was referred to speech therapy. Over the following two years, the patient improved steadily with speech therapy until she became infected with SARS-CoV-2, at that point she lost her voice completely. Interestingly, the patient's vocal cord nodules seemed to have resolved during the VCP. This finding could be a result of her inability to use her voice while her vocal cords were paralyzed, effectively forcing her to observe vocal rest, or the nodules persisted but were masked by edema due to the infection. The patient recovered without any medical or surgical treatment for VCP. Since her recovery, the patient has had complete return of vocal cord mobility and vocal cord nodules.

## 3. Discussion

Trauma and malignancy are two well understood causes of VCP that account for 28.89% and 31.11% cases, respectively [3]. A large portion of cases (29.8–65.7%) are reported as idiopathic in etiology [8]. Many speculate that viral etiologies could account for a significant portion of the cases currently considered idiopathic [9]. Retrospective studies have supported this and found that a high number of upper respiratory infections correlated with idiopathic cases of VCP [10, 11]. Other studies have criticized this conclusion, remarking on the lack of an elucidated causal relationship and a small sample size [8]. In the study conducted by Rubin et al., viral infection was only found to be present in 8% of idiopathic VCP cases [8]. Thus, the speculation that a viral etiology is the culprit behind idiopathic VCP remains unclear. In either case, viruses are undoubtedly capable of causing VCP, and with the pervasiveness of the COVID-19 pandemic, there is mounting evidence that SARS-CoV-2 could be one such virus.

However, most cases of SARS-CoV-2 present asymptomatically or with mild symptoms, and the virus is capable of progressing to respiratory failure and other serious disease states. At times, the virus has been shown to produce neurological symptoms like anosmia, Guillain–Barre, Bell's palsy, and encephalopathy [12–16]. In addition, rare cases have been reported of COVID-19-related VCP [7, 17–20]. These patients typically develop vocal cord dysfunction at the onset of a COVID-19 infection concurrent with the development of traditional viral symptoms. Some patients may develop vocal cord dysfunction and test positive for COVID-19 as a part of their clinical workup. The vocal cord dysfunction is usually transient and does not require intervention, yet some cases have required various degrees of treatment [18, 19].

Viral infections cause VCP through a variety of mechanisms. Viruses with a propensity for infecting nerves, like VZV and HSV, are capable of directly causing VCP [21, 22]. Given a meager yet growing prevalence of SARS-CoV-2 neurotropic effects, a direct infection of the laryngeal nerves is not certain and the exact mechanism behind neuropathic symptoms is mostly speculation. Given the patient's significant history of prior vocal cord dysfunction, the edema associated with a SARS-CoV-2 infection and shown in Figure 1 could result in soft tissue swelling capable of nerve compression and a transient VCP. The virus might also precipitate an exacerbation of muscle tension dysphonia, which is reportedly capable of mimicking VCP [23]. The patient's VCP could be the result of direct infection of the laryngeal nerves by SARS-CoV-2, given that other cases of VCP have presented in patients with no history of vocal cord dysfunction and minimal edema of the vocal cords, indicating a more direct mechanism. Irrespective of the mechanism, SARS-CoV-2 seems to be capable of causing VCP. This finding could have substantial implications given the prevalence of the coronavirus worldwide. The large number of asymptomatic cases caused by the corona virus could have allowed it to remain an undetected cause of VCP, and severe cases of COVID-19 that develop VCP after requiring intubation could be mistaken as having VCP caused by trauma rather than the virus itself.

## 4. Conclusion

This report presents one of the few documented occurrences of SARS-CoV-2-related VCP in the absence of intubation. The patient is the youngest in the current literature. Having excluded trauma and other known causes with CT scanning, the SARS-CoV-2 virus is the likely cause of the patient's paralysis due to its correspondence with the time of onset. This finding contributes to the emerging variety of neuropathic effects found in SARS-CoV-2 infections and the idea that viral infections are a significant etiology of VCP.

## Data Availability

Data used to support the findings in this study are from public resources and can be found within the articles cited in the reference section.
